# Creation of Long-Term Physical Stability of Amorphous Solid Dispersions N-Butyl-N-methyl-1-phenylpyrrolo[1,2-a]pyrazine-3-carboxamide, Resistant to Recrystallization Caused by Exposure to Moisture

**DOI:** 10.3390/ma18010203

**Published:** 2025-01-06

**Authors:** Vladimir B. Markeev, Evgenia V. Blynskaya, Konstantin V. Alekseev, Vladimir L. Dorofeev, Anna I. Marakhova, Alexandre A. Vetcher

**Affiliations:** 1 Federal Research Center for Innovator and Emerging Biomedical and Pharmaceutical Technologies, 8 Baltiyskaya Str., 125315 Moscow, Russia; markeev_vb@academpharm.ru (V.B.M.); blynskaya_ev@academpharm.ru (E.V.B.); alekseev_kv@academpharm.ru (K.V.A.); info@academpharm.ru (V.L.D.); 2 Institute of Pharmacy and Biotechnology, Peoples’ Friendship University of Russia n.a. P. Lumumba (RUDN), 6 Miklukho-Maklaya Str., 117198 Moscow, Russia; agentcat85@mail.ru; 3 Institute for Bionic Technologies and Engineering, I.M. Sechenov First Moscow State Medical University, 2-4 Bolshaya Pirogovskaya Str., 119991 Moscow, Russia

**Keywords:** amorphous solid dispersion, active pharmaceutical ingredient(s), recrystallization

## Abstract

Amorphous solid dispersion (ASD) technology is often used as a promising strategy to improve the solubility of active pharmaceutical ingredients (APIs). ASDs allow APIs to be dispersed at the molecular level in a polymer carrier, destroying the crystalline structure of the APIs and, thanks to the polymer, providing long-term supersaturation in solution. However, stability issues are an obstacle to the development of new medications with ASD. In addition to the molecular mobility at elevated temperatures leading to the crystallization of APIs, moisture affects the physical stability of ASD, leading to fractional separation and recrystallization. N-butyl-N-methyl-1-phenylpyrrolo[1,2-a]pyrazine-3-carboxamide (GML-3) is an original API with both anxiolytic and antidepressant activity, but its insolubility in water can negatively affect (influence) bioavailability. Our study aims to create ASD GML-3 with moisture-resistant polymers (Soluplus^®^, HPC) and assess the stability of the amorphous state of ASD after storage in high humidity conditions. As a result, HPC Klucel^TM^ FX was revealed to be more stable than the brand, providing a high level of API release into the purified water environment and stability after 21 days (3 weeks) of storage in high humidity conditions.

## 1. Introduction

Up to 40% of the compounds on the market and more than 70% of candidate compounds for active pharmaceutical ingredients (APIs) have poor solubility in water, falling into Class II and IV of the Biopharmaceutical Classification System (BCS). This may negatively affect their bioavailability and complicate the development of new medications [1,2,3,4].

According to the Noyes–Whitney equation, the solubilization of poorly soluble compounds in water is possible by increasing the contact area between the API and the aqueous medium or by achieving an oversaturated state in solution [5,6,7,8]. The surface area can be increased through micronization, while the oversaturated state of the API is achieved by modifying the crystal structure of the particles, which includes the creation of polymorphic API forms, co-crystallization, and the production of solid dispersions (SD) [9,10,11]. Among the methods for altering the crystal structure of the API is the creation of solid dispersions, which allows for the simultaneous reduction in particle size and alteration of the crystallinity level of the API [12,13,14,15].

Solid dispersions are composites in which the API is dispersed in a polymer carrier, exhibiting either eutectic behavior or complete amorphousness.

On the one hand, supersaturation in solution is achieved due to the fact that SD improves the wettability of API in an aqueous environment. On the other hand, in most cases, it is possible to convert the API inside SD into a more soluble amorphous form, which demonstrates significantly superior dissolution kinetics compared to its crystalline counterparts [16,17,18].

Various technologies are used to produce SD, which can be divided into two main groups: dissolution methods (solvent removal, electrospinning, supercritical anti-solvent, spray drying, etc.) and heating (hot-melt extrusion, melting, melting-mixing, etc.).

Depending on their composition, SDs are divided into three generations [8]. SDs with crystalline carriers, so called “eutectic mixture” belong to the first generation [19,20,21,22]. Amorphous solid dispersions (ASD), where the API is completely amorphous in a non-crystalline polymer carrier, represent the second generation [18,23,24,25,26]. Their advantage is the possibility of converting the API to a more soluble amorphous state with the appearance of “parachute” and “hover” effects, which ensure API oversaturation in solution [26]. ASD of the third generation contains a surfactant [1,27].

Meng et al. developed a new classification that more accurately describes the crystallinity of the API and polymer within the SD, dividing SDs into 6 classes based on the API-polymer state: C-C (crystalline API in crystalline polymer), C-A, A-C, A-A, M-C, and M-A [28]. Second generation SD (ASD) corresponds to classes A-A (amorphous API in amorphous carrier) and M-A (amorphous API dispersed at molecular level in amorphous carrier with no residual crystalline structures).

The APIs in the ASD tend to change to a more stable state during storage, leading to a potential decrease in the dissolution rate of the API and consequently a decrease in bioavailability [4]. Thus, the physical stability of the ASD is an important parameter in drug development and should be monitored due to its strong influence on therapeutic efficacy [29,30,31].

In addition to the proper choice of carrier polymer and temperature, humidity has an effect on the stability of the ASD, lowering the glass transition temperature and causing a plasticizing effect that increases the molecular mobility of the ASD. Absorbed water can also potentially interfere with API-polymer interactions, competing with the polymer to form hydrogen bonds.

ASDs are usually stored at room temperature, although there are no established protocols for predicting and determining their physical stability. The 40 °C/75% relative humidity (RH) are the recommended storage conditions according to the International Council for Harmonisation (ICH) (FDA Centre for Drug Evaluation and Research, Stability Testing of New Drug Substances and Products Q1A). The FDA recommendations are aimed at evaluating the stability of APIs and tablets during storage, as ASD is often used in tablet manufacturing. In different studies, in addition to different temperatures and humidity, different test periods are used: 7, 21 days, 1–3 months, (sometimes) half a year, and/or a year. We decided to study the stability of ASD at a temperature of 40 °C and a relative humidity of 75% for 21 days. Such parameters are often used in stability studies and make it possible to predict the stability of a future drug at the development stage.

**Figure 1:**
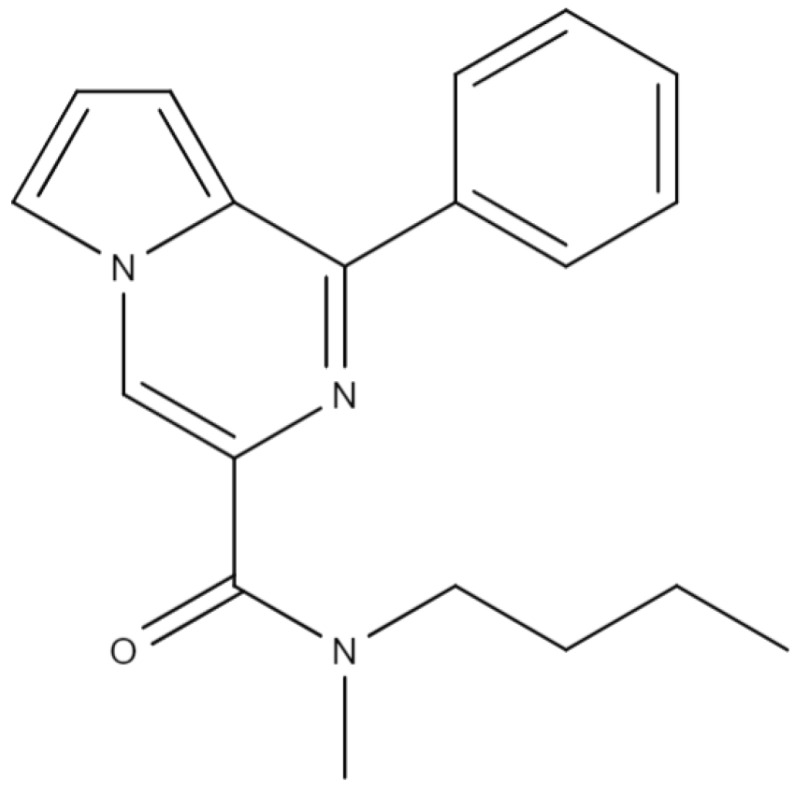
Structural formula of GML-3.

The original active pharmaceutical ingredient N-butyl-N-methyl-1-phenylpyrrolo[1,2-a]pyrazine-3-carboxamide (GML-3) (Figure 1) synthesized in the Federal Research Center for Innovator and Emerging Biomedical and Pharmaceutical Technologies (Russia, Moscow) has a high affinity (Ki = 5.3 × 10^−7^ M) with the 18 kDa mitochondrial translocator protein (TSPO) [32,33]. GML-3 shows pronounced antidepressant and anxiolytic activity when tested on animals; however, hydrophobicity and crystallinity are problems in the development of a dosage form for oral use [34]. GML-3 is practically insoluble in water (less than 1:10,000). In previous articles, we addressed the issue of improving the solubility of API GML-3 by creating ASD with povidone (PVP) and Soluplus^®^ [35]. The main problem of PVP was the need to use a high amount of polymer to inhibit the crystallization of GML-3 in SD during storage under conditions of “accelerated aging” at elevated temperatures [35]. Soluplus^®^ showed rather high stability during storage, at a concentration of 1:5 or more with the presence of weak API-polymer interaction. According to Patel and Serajuddin, PVP with different molecular weights is highly hygroscopic, reaching from 22 to 42% humidity by weight when stored under different conditions [36]. As an example of the effect of humidity directly on ASD, in the study by Karon et al., sulfadimidine ASD/Soluplus^®^ had a residual moisture content of 3.4% moisture, while sulfadimidine-based ASD/PVP loses 11.6% moisture [37]. Due to the high hygroscopicity of PVP and poor stability of SD with GML-3, only the 1:15 ratio gave stability in the absence of moisture, making us reconsider the creation of SD with GML-3 taking into account the influence of moisture on the stability of ASD. This is especially important for Soluplus^®^, which formed ASD in our previous study at a ratio of 1:5, which, due to the high viscosity of the solution, was a problem in the development of GML-3 tablets. Thus, it is important to reduce the Soluplus^®^ requirement in ASD GML-3. Another obstacle to the development of an oral dosage form of GML-3 may be the high polymer content in SD formulations, which negatively affects the disintegration capabilities of tablets and complicates the process of their production. Taking into account these facts, as well as the thermolability of API GML-3 and its solubility in ethanol, the aim of our study was to develop ASD of GML-3, providing long-term stability under conditions of increased humidity and temperature, and improve the level of API release into the medium of purified water.

The objects of study were API GML-3, SD GML-3 with PEG, Soluplus^®^, and hydroxypropyl cellulose (HPC) obtained by the solvent removal method (the simplest in technological terms). Ethanol served as the solvent. HPC and Soluplus^®^ were well soluble in ethanol and relatively stable when exposed to moisture, and semi-crystalline PEG served as a comparison polymer that did not form ASD and was not stable when exposed to moisture. Methods for obtaining SD by heating were not used in extrusion process with polymer due to the risk of darkening the GML-3. The degradation of API slowly starts after overcoming the melting temperature. SD were obtained in API/polymer ratios = 1:2 and 1:4. Such API/polymer ratios minimize the technological risks associated with the further development of GML-3 tablets. The stability test was performed after storage under the parameters of recommended storage conditions according to ICH harmonization guidelines −40°C/75%RH relative humidity. This method allows us to evaluate the stability of ASD without prolonged storage tests (1–2 years).

## 2. Materials and Methods

### 2.1. Materials

API: GML-3 (Federal Research Center for Innovator and Emerging Biomedical and Pharmaceutical Technologies, Moscow, Russia). Release series: 14122023. Date of manufacture: 14 December 2023. Figure 1 shows the structural formula of GML-3.

Excipients (Es): Hydroxypropyl cellulose (Klucel^TM^ LF, Klucel^TM^ EF) were obtained from (Ashland GH Inc., Wilmington, DE, USA). Graft copolymer polyvinyl caprolactam-polyvinyl acetate polyethylene glycol (Soluplus^®^) was obtained from (BASF AG, Ludwigshafen, Germany). PEG 4000 MW, powder, was obtained from (Merck Lifesciences, Budapest, Hungary).

Solvents: distilled water was obtained on the PE-2205 apparatus (Ecroskhim Ltd., St. Petersburg, Russia), and ethanol HP (99.5%, 0.005% maximum water) was obtained from (Merck KGaA AG, Darmstadt, Germany).

### 2.2. Methods

#### 2.2.1. Generation of SD/ASD GML-3

GML-3 (1.00 g) was loaded into a closed container (50 mL) and dissolved in 99.5% ethanol (35.00 g). The dissolution was carried out using a magnetic stirrer PE-6100 (Ecroskhim Ltd., St. Petersburg, Russia). Polymer (HPC, Soluplus^®^, PEG 4000) was gradually added to the resulting solution and mixed until a homogeneous transparent solution was obtained. The resulting solution was dried at 55 °C for 24 h.

#### 2.2.2. Differential Scanning Calorimetry (DSC)

Thermal analysis was carried out using a DSC 204 HP Phoenix^®^ differential scanning calorimeter (NETZSCH, Selb, Germany) at ambient pressure. The instrument was pre-calibrated for temperatures and enthalpies of phase transitions of pure (99.99+%) standard substances in compliance with the ASTM Practices E 967 and E 968: H_2_O, benzoic acid, In, Sn, Bi, Pb, and Zn. The RMSDs for temperature and heat effect determination were 0.2 °C and 5%, respectively. The experimental data were processed with the NETZSCH Proteus^®^ Software (version 7.1) according to ASTM and ISO 11357-1 [38,39].

Samples weighing 1.00–3.00 mg were ramped from 10 to 150 °C with a heating rate of 5 °C/min under dry synthetic air (O_2_ (20.9 ± 0.5)%, N_2_ (79.1 ± 0.5)%, with CH_4_, CO, CO_2_ < 0.005%) flow of 40 mL/min in aluminum crucibles with pierced lids (V = 25 mm^3^, d = 5 mm).

#### 2.2.3. X-Ray Powder Diffraction (XRPD)

The X-ray powder diffraction (XRPD) spectra of SD GML-3 were recorded on a desktop X-ray diffractometer, a Miniflex 600 (Rigaku Corp., Tokyo, Japan). The measurements were carried out using Cu Ka radiation at 40 kV and 40 mA in the range of 2θ 5–65° with a scanning speed of 4°/min and a step size of 0.05°.

#### 2.2.4. Dissolution Test

This test was carried out according to European Pharmacopoeia 10 edition (2.9.3. Dissolution test for solid dosage forms, Physical Tests (711) The United States Pharmacopeia, Volume 4). The medium of dissolution was distilled water with a volume of 900 mL. Dissolution medium temperature was 37.0 ± 0.5 °C with a sampling time of 1, 3, 5, 10, 15, 30, 45, 60 min; we replenished the medium after each sampling (10 mL). The absorption was determined using a spectrophotometer PE-5400UF (Ecroskhim Ltd., St. Petersburg, Russia) at a wavelength of λ = 256 nm.

In order to simulate the dissolution kinetic of GML-3 from SD, the Korsmeyer–Peppas model was used [40]:

*M_t_*/*M*_∞_ = *k·t^n^*

where *M_t_* is the amount of dissolved GML-3 in any time *t*, *M_∞_* is the amount of dissolved GML-3 at infinite time, *k* is the release rate constant and *n* is the release exponent. Release exponent *n* provides information on the API release mechanisms involved while kinetic constant *k* includes structural and geometric characteristics of the solid dispersion.

#### 2.2.5. Accelerated Aging Stability Tests

ASD GML-3 powder samples were accurately weighed into clear glass vials under ambient conditions. ASD powder was also placed in open dishes inside climate chambers (HPP410 eco, Memmert, Germany) at 40 °C/75%RH conditions relative humidity for 3 weeks.

## 3. Results

**Figure 2:**
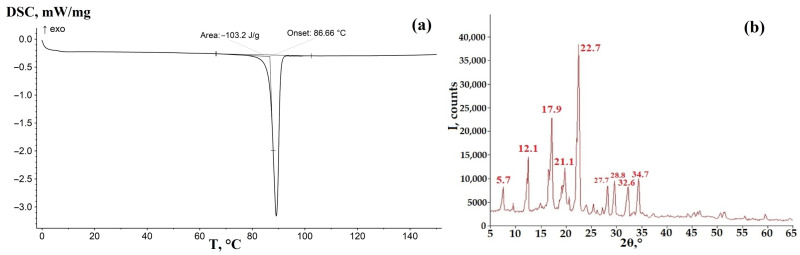
DSC (**a**) and XRPD (**b**) data for the API GML-3 used to create the ASD.

DSC and XRPD data for GML-3 showed good agreement with the literature data concerning the melting point (88.66 °C) and XRD peaks (Figure 2) [41].

According to PXRD, under normal conditions, the compound GML-3 used for ASD preparation is a polycrystal with characteristic peaks for polymorphic Form I at 5.7°, 12.1°, 17.9°, 21.1°, and 22.7°, which is in agreement with the literature data [42]. GML-3 is completely in crystalline state without amorphous structures. This is confirmed by the absence of halo on PXRD (peaks wider than 3°), which is one of the characteristics of amorphous substances. According to the DSC data, GML-3 starts to melt at 88.66 °C (Figure 2a). The difference with literature data of 1.5 °C may be due to the peculiarities of instrument calibration. The energy released was 103.2 J/g, versus 95.56 J/g—the difference indicates only distinction filling of the crucible surface. It is difficult to determine the lattice type of Bravais API, due to the impossibility of growing a single crystal.

### 3.1. ASD GML-3 Crystallization Analysis with Soluplus^®^

**Figure 3:**
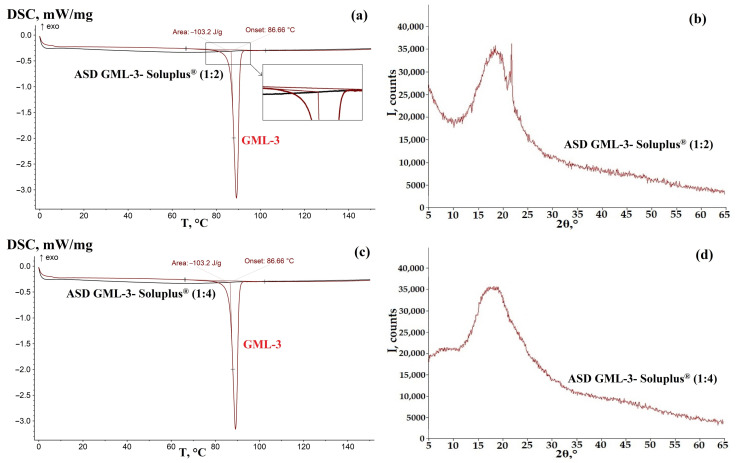
DSC (**a**,**c**) and XRPD (**b**,**d**) data for ASD GML-3: Soluplus^®^.

The feasibility of using Soluplus^®^ polymer as a basis for creating a stable ASD system is due to its lower hygroscopicity compared to PVP, PEG, and PVP VA64, which is a copolymer of N-vinylpyrrolidone with vinyl acetate [30,36]. DSC analysis of GML-3—Soluplus^®^ ASD samples at 1:2 and 1:4 ratios showed a complete absence of crystalline structures, indicating that GML-3 is completely amorphous within the ASD (Figure 3a,c). There may be crystals smaller than 50 nm, which are poorly identifiable by DSC.

In contrast, XRPD analysis revealed crystallinity for Soluplus^®^ at a 1:2 ratio (Figure 3b). There is a pronounced peak at 21.7°, which is the most intense peak characteristic of GML-3. There are also distinguishable peaks at 17.9° and 12.1°, which are less identifiable due to the increasing first amorphous polymer halo. It is worth noting the presence of the first amorphous halo (its decreasing part 5°–11°), which is characteristic of Soluplus^®^. As shown in an earlier study, its pronounced presence indicates the absence of a weak molecular bond between the polymer and GML-3 [35]. The presence of a weak bond between the polymer and API (fading of the first halo) usually indicates an improvement in the kinetics of API dissolution. Thus, at a ratio of 1:2, we have a C-A system (according to Meng), a weakly crystalline structure where API crystals are dispersed in the amorphous polymer. It is concluded that it was not possible to achieve complete inhibition of API crystallization and obtain ASD.

At a ratio of 1:4, the situation changed. The DSC data remained the same—a completely amorphous structure with no traces of API GML-3 melting (Figure 3c). The XRPD data became characteristic of an amorphous substance (Figure 3d). Also, the XRPD data recorded a decrease in intensity (smoothing) of the first amorphous Soluplus^®^ halo polymer, indicating a weak API-polymer interaction (Figure 3d). Thus, a ratio of 1:4 is the minimum ratio to obtain ASD (2nd generation, Meng class A-A).

### 3.2. Analysis of the Crystallinity of SD GML-3 with PEG

**Figure 4:**
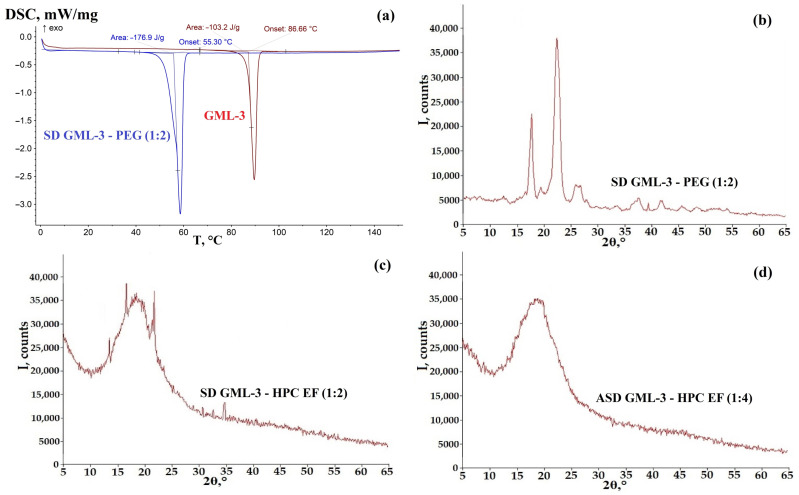
DSC data for SD GML-3: PEG (1:2) (**a**), XRPD data for SD GML-3: PEG (1:2) (**b**), XRPD data for SD GML-3: HPC EF (1:2) (**c**), ASD GML-3: HPC. EF (1:4) (**d**).

Strongly hygroscopic, semi-crystalline PEG usually forms “eutectic mixtures” [40]. SD GML-3 (1:2) with semi-crystalline PEG 4000 was characterized by two high-intensity peaks and two medium-intensity peaks in the XRD data (Figure 4b). For SD GML-3–PEG (1:4), the XRPD data were similar to those for the 1:2 ratio. The DSC data are characteristic of a fully crystalline SD–eutectic mixture with a single melting peak (Figure 4a).

The resulting SD has a melting point of 55.3 °C with 176.9 J/g energy release, which is typical of a eutectic mixture (Figure 4a). The melting point roughly corresponds to the arithmetic mean of the Tm of the components (4 degrees lower); slightly lower values may be due to the prevalence of PEG in the SD composition. In this case, the creation of a crystalline type SD–1st generation, C-C system is evidenced by the single melting point of the components and the change in the XRPD peaks with respect to GML-3 (any further increase in the polymer does not yield XRPD changes). The two main XRPD peaks correspond to van Hecke and Benani’s data for PEG 4000 [43]. In the work of Heike Bley, Bernd Fussnegger, and Roland Bodmeier, two main XRPD peaks and three peaks of average intensity (40°) corresponded to the data for PEG, which indirectly suggests that SD is obtained without inclusions of GML-3 crystals [44]. In this case, PEG is taken to compare ASD with SD in terms of the effect of crystallinity on stability when exposed to moisture. PEG has a limited solubilization effect, improving wetting on the API surface, but cannot withstand exposure to moisture, leading to phase separation.

### 3.3. Analysis of the Crystallinity of ASD GML-3 Using HPC

The prospect of using HPC is due to its greater stability of the amorphous state when exposed to moisture [45]. We used two brands of HPC: Klucel^TM^ LF (molecular weight 95,000 g/mol) and Klucel^TM^ EF (molecular weight 80,000 g/mol). Some researchers theorize that decreasing the molecular weight of HPC improves the stability and suppresses the molecular mobility of API molecules, preventing α-relaxation in the system [46]. However, the use of grades like SSL (40,000 g/mol) and UL (20,000 g/mol) entails problems in the development of tablets and capsules, which are the major medicines with SD. Some of the few medicines on the market with HPC are Samsca^®^ (API Tolvaptan is a selective vasopressin V2-receptor antagonist), available since 2018 under FDA approval, and Eucreas^®^ Galvumet^TM^ (API Vildagliptin/Metformin HCL) for the therapy of type II diabetes, manufactured by Novartis since 2007 under EMA approval [12,47].

Small dosages do not allow for sufficient viscosity when using low-molecular-weight HPCs, so either other polymers such as water insoluble Eudragit^®^ are added to the SD formulation or the molecular weight of the HPC is increased. For this reason, we have chosen HPCs with a medium molecular weight. They are well-soluble in alcohol, as is GML-3, which allows the use of a solvent removal method. It is also worth noting that in Petkov’s study for glibenclamide ASD, a decrease in m.m. contributed only to a greater supersaturation of the API in solution, but not to stability or less hygroscopicity, which suggests that there is no general relationship between m.m. and stability due to the influence of the structure of the API itself and the methods of preparation of ASD [48].

**Figure 5:**
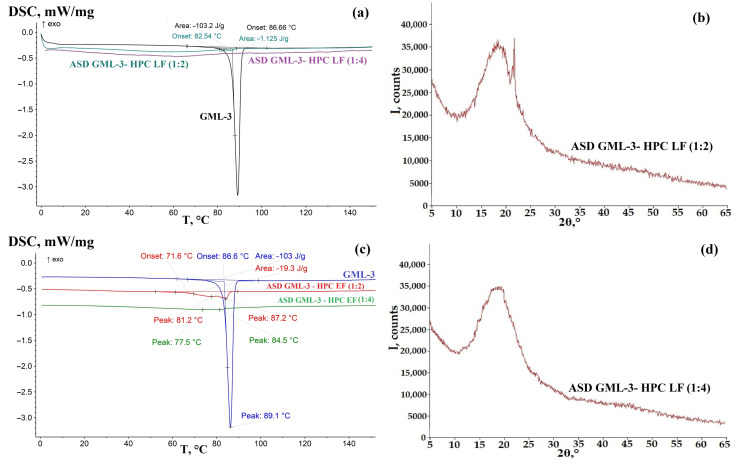
DSC data for SD GML-3—HPC LF (**a**) and ASD GML-3—HPC EF (**c**), XRPD data for SD GML-3—HPC LF (1:2) (**b**), ASD GML-3—HPC LF (1:4) (**d**).

For HPC EF at a 1:2 ratio, the thermogram captures “steps”—two smeared half peaks, at 81.2 °C and a more pronounced one at 87.2 °C with a total energy expenditure of 19.3 J/g (Figure 5c). The more pronounced peak roughly corresponds to the melting of the residual GML-3 crystals. The melting peak is conditionally pronounced, which may indicate residual crystallinity and loss of homogeneity by the mixture. XRPD data indicate the presence of GML-3 crystals in samples with HPC EF (1:2) (Figure 4d). At a 1:4 ratio, there is little or no evidence of crystallinity according to XRPD and DSC data (Figure 4d and Figure 5c). A non-intensive process is present when ASD heating proceeds prior to the onset of crystallization—the formation of API-rich zones as the first poorly identifiable endoderm at 75.5 °C and 84.5 °C (Figure 5c). The total process energy in which GML-3 partially crystallized at the level of residual crystallinity, namely the zones with poorly structured crystals due to polymer interference and the melting zone of relatively large GML-3 crystals, was 19.3 J/g and very extended in time. Thus, with HPC EF at a ratio of 1:4, it can be considered an ASD system of type A-A.

For LF-grade HPC, the situation was different. At a 1:2 ratio, residual crystallinity in the form of melting at 82.54 °C with 1.125 J/g heat absorption was observed (Figure 5c). In the article by Rashid et al., a similar pattern was observed when analyzing the mechanical mixture of ezetimibe and low-viscosity HPC, but, in our case, the peak is weakly pronounced, from which it can be concluded that after recrystallization of GML-3 and polymer, a homogeneous mixture with little signs of residual crystallinity was obtained [49].

X-ray diffraction confirmed the presence of crystalline structures in the GML-3: HPC LF (1:2) ASD as the main peak of GML-3 at 22.7 °C (Figure 5b). Thus, at a 1:2 ratio, HPC LF is unable to completely prevent crystallization of GML-3 during solvent removal during ASD creation. At a 1:4 ratio, no glass transition or melting processes were detected on the thermogram, and the XRD was identical to the polymer (Figure 5a,b).

Thus, M-A systems were formed with Soluplus^®^ and HPC LF polymers at 1:4 ratios. HPC EF forms an A-A system at a 1:4 ratio.

### 3.4. Evaluation of the Effect of Humidity on the Crystallinity of GML-3 in ASD

**Figure 6:**
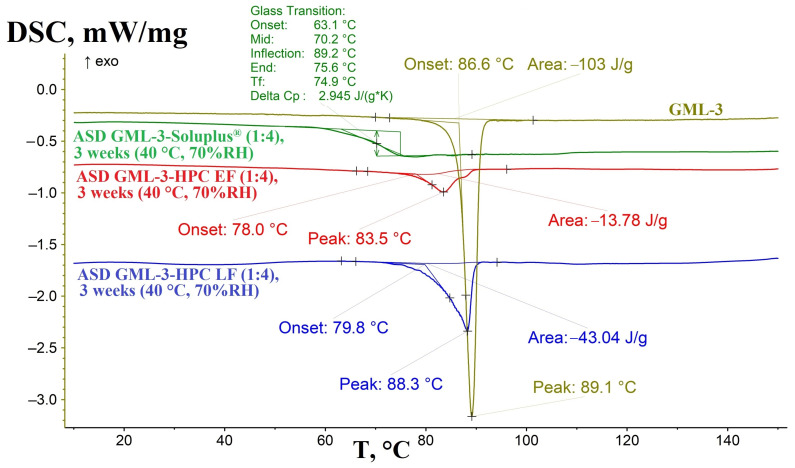
DSC data for GML-3 (light green) GML-3, ASD GML-3:Soluplus^®^ (1:4) (green) and ASD GML-3:HPC (1:4) EF (red) and LF (blue) after 3 weeks of storage.

After storage at 40 °C and 75%RH of the amorphous solid dispersions, the samples were examined by DSC for residual crystallinity (Figure 6). The Soluplus^®^ polymer showed a high level of crystallization inhibition by API GML-3 for 21 days (3 weeks) under “accelerated aging” conditions at elevated humidity and a 1:4 (M-A) ratio. Upon heating the ASD GML-3: Soluplus^®^ (1:4) sample, Soluplus glass transition was revealed by the DSC method, which starts at 63.1 °C and ends at 75.6 °C with a heat absorption of 2.945 J/(g*K). Slight fluctuations in the thermogram after glass transition were also seen, until a temperature of 89.2 °C was reached, which corresponds to the glass transition temperature of API GML-3. These processes may be related to the molecular mobility of API GML-3. These processes are characteristic of glassy (amorphous) substances. It is concluded that, in this case, there is a 2nd generation M-A class Meng system and high stability of ASD with Soluplus^®^.

**Figure 7:**
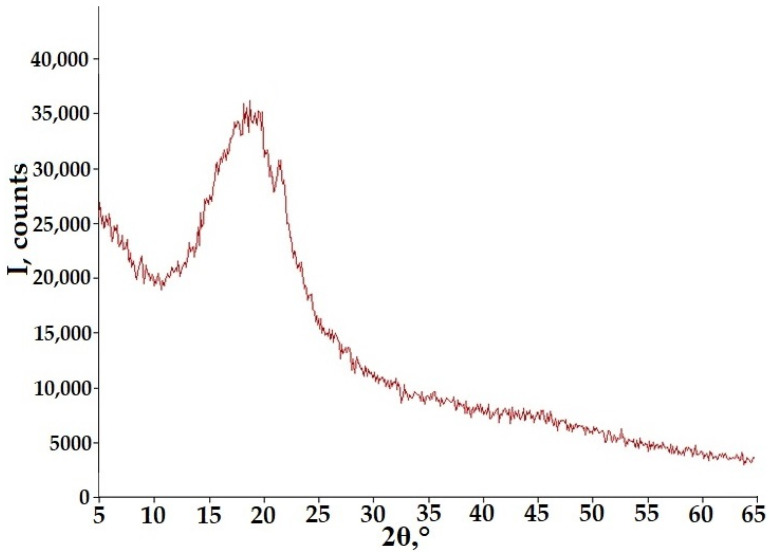
XRPD for ASD GML-3: HPC EF (1:4) after storage.

The thermogram for ASD GML-3 HPC after storage changed strongly compared to the original ASD. According to the DSC data, the initially more amorphous ASD GML-3 HPC LF (1:4) acquired a strong crystallinity characteristic of GML-3 crystals during storage. The onset of melting was recorded at 79.8 °C, with a peak at 88.3 °C and an absorption of 43.4 J/g, which is more characteristic of GML-3. HPC LF did not inhibit the crystallization of GML-3, with a C-A system obtained during storage, with a large degree of crystallization of GML-3. According to the XRPD data for ASD GML-3: HPC EF (1:4) after storage, the presence of a smeared peak of 22.7°, which is characteristic of GML-3 crystals, was detected (Figure 7). Its relatively small intensity is associated with the fact that GML-3 crystals did not have time to grow to a significant size.

HPC XF, possessing a lower molecular weight compared to LF, more effectively inhibited the crystallization of GML-3. The residual crystallinity peak, at 84.5 °C in the original sample, was poorly discernible, which may indicate the presence of poorly structured residual zones with elevated GML-3 content and the almost complete absence of residual crystalline structures. Despite the presence of this crystallization precursor, after 3 weeks of storage at elevated humidity, an almost imperceptible secondary “step” of the crystallization precursor was recorded, indicating the presence of the A-A system.

Thus, it was found that ASD with Soluplus^®^ is completely stable during storage in high humidity conditions, HPC XF is generally stable, which confirms the theory of better stability of lower molecular weight HPC grades. Thus, ASD HPC XF (80,000 g/mol) despite evidence of localized residual structures (A-A) is more stable than GML-3 (M-A) dispersed in HPC LF (95,000 g/mol) completely at the molecular level. ASD with HPC LF (1:4) rapidly crystallized to form GML-3 crystals during storage.

### 3.5. Comparative Dissolution Kinetics of SD GML-3:PEG, ASD GML-3:Soluplus^®^ and ASD GML-3:HPC

**Figure 8:**
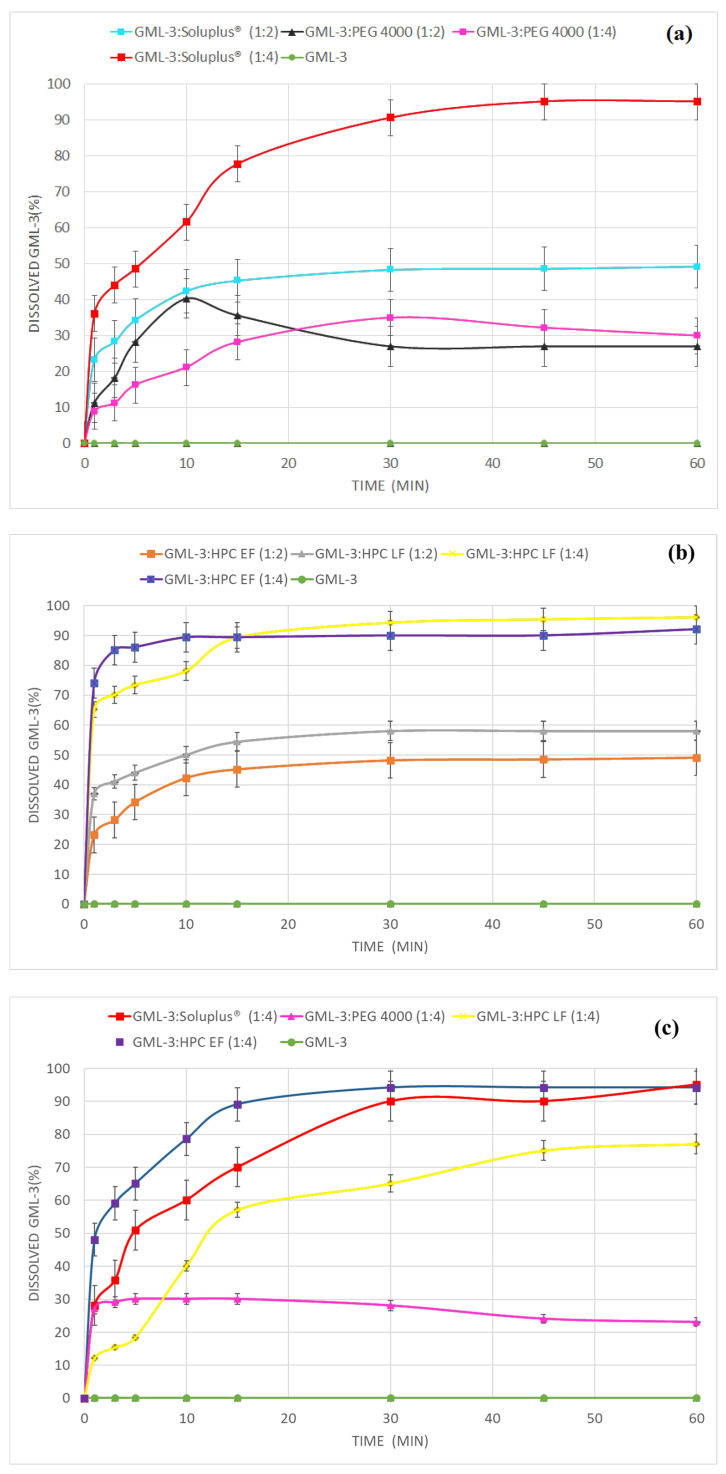
Release kinetics of GML-3 from SD GML-3 with PEG 4000 and Soluplus^®^ (**a**), HPC (**b**) and after ASD after storage for 3 weeks at 40 °C and 75%RH (**c**).

According to the “Dissolution test for solid dosage forms” performed for all samples, the effect of ASD on the release rate of GML-3 was detected (Figure 8a,b).

SD GML-3:Soluplus^®^ (1:4) released 91% of GML-3 in 30 min, and by 60 min the release reached 95% (Figure 8a). At a 1:2 ratio, ASD was already absent, causing only 47% release of GML-3 by 30 min. From the crystalline SD of GML-3:PEG, the release of GML-3 was 30% and 40% at API-polymer ratios of 1:2 and 1:4 (Figure 8a). There was also a precipitation effect from the solution, resulting in 25% and 30% of GML-3 in solution by 60 min. This is because the 1st generation SD improves API wettability and reduces API agglomeration during dissolution. Thus, PEG is unable to provide supersaturation of API in solution and from slowing down the process of concentration decline.

When ASD GML-3:HPC LF (1:4) and ASD GML-3:HPC EF (1:4) were examined, an increased level and rate of release was observed for ASD (up to 90% by 15 min) (Figure 8b). For the 1:2 concentration, less than 60% was released due to the residual crystallinity of GML-3 (Figure 8b).

### 3.6. Comparative Dissolution Kinetics of SD GML-3:PEG, ASD GML-3:Soluplus^®^ and ASD GML-3:HPC After Storage Under High Humidity Conditions

To evaluate the stability of ASD, all samples of class A-A, M-A, and C-C were analyzed for changes in GML-3 release after storage. Storage for 3 weeks at 40 °C and 75%RH of ASD GML-3:Soluplus^®^ (1:4), ASD GML-3:HPC LF (1:4) and ASD GML-3:HPC EF (1:4), and SD GML-3:PEG (1:4) (Grade C-C) revealed the effect of humidity on the stability of the amorphous state within the ASD (Figure 8c). SD PEG was investigated for comparison as a fully crystalline SD. SD PEG delaminated after storage, indicating its instability as an inhibitor of the crystallization of GML-3 molecules and the solubility dropped to 27.2% with a decreasing trend due to precipitation of GML-3 and not being able to PEG. The delayed dissolution of SD GML-3:PEG (1:4) is associated with a high content of PEG in SD. For this reason, the rate of PEG dissolution further limited the kinetics of GML-3 dissolution. ASD GML-3:Soluplus^®^ (1:4) generally maintained the same release rate of −u ≥ 90% by 30 min. This may indicate the stability of ASD GML-3:Soluplus^®^ (1:4) during storage.

ASD GML-3:HPC EF (1:4), after “accelerated aging” at 40 °C and 75%RH, achieved 90% release by 15 min (vs. 10 min without “accelerated aging”) and without a drop in total release (difference within the error zone). ASD GML-3:HPC LF (1:4) exhibited a rather severe slowing of release after storage, reaching a level of 86.2% release only by 60 min. This indicates the crystallization of GML-3, and the release is provided more with the properties of the polymer, which prevents GML-3 from precipitating. This result can be considered as negative, because the strong influence of external factors on the solubility of GML-3 will not allow researchers to achieve repeatable results and stable results for the release of GML-3.

The Korsmeyer–Peppas equation is commonly accepted to describe API diffusion from SD, e.g., by Vasconcelos et al., who evaluated the diffusion of API resveratrol from the third generation ASD combining Soluplus^®^ and poloxamer 407 [50]. As a result, the authors identified a nonlinear (anomalous) diffusion of API solutions from ASD, which is not described by Fick’s law. Milovanovic et al. used ASD to increase the dissolution rate of carvedilol from ASD obtained with Soluplus^®^, Eudragit^®^ (synthetic copolymer derived from esters of acrylic and methacrylic acid), and hydroxypropyl methylcellulose acetate succinate (HPMC-AS) [51]. Analysis of API carvedilol release from TD with HPMC-AS also revealed abnormal diffusion of API carvedilol from SD. SD carvedilol with Eudragit^®^ demonstrated less limited and faster API release of carvedilol compared to the other two SD. Gong et al. demonstrated that the Korsmeyer–Peppas model is well suited for describing the dissolution rate of API indomethacin from ASD with hydroxypropylmethylcellulose, and it was also reported that n is 0.54 [52]. The correlation coefficients of the dissolution of GML-3 from SD, according to the Korsmeyer–Peppas model, limited to the description of the first 60% of the release, are presented in Table 1.

All formulations in Table 1 had a total release rate of more than 60%. All compositions shown in the table had a value of n ˂ 0.45 (diffusion according to Fick’s law). The highest value of the release rate constant before “accelerated storage” was observed in SD GML-3:HPC EF (1:4) k = 0.842. For this SD, high values persisted after 3 weeks (40 °C and 75%RH) k = 0.576. For ASD GML-3:Soluplus^®^ (1:4) the values of k before/after storage are the same (k = 0.358/0.350). A significant decrease is recorded in ASD GML-3:PCL F (k = 0.627/0.155), which confirms the negative effect of recrystallization inside SD on the solubility of GML-3.

## 4. Conclusions

The results of this study indicate that the creation of ASD GML-3 with Soluplus^®^ and Klucel^TM^ XF at a ratio of API: polymer = 1:4 will produce an ASD that is stable when exposed to moisture. ASD GML-3:Soluplus^®^ forms a class M-A system that provides complete inhibition of crystallization of GML-3 molecules, stable against moisture during 3 week storage and complete dissolution of GML-3 in water. ASD GML-3:HPC EF, which is an A-A system, with minor traces of crystallization, does not undergo further significant crystallization during storage at 40 °C and 75%RH, giving long-term stability of the amorphous state of GML-3 with a high release rate of more than 90%. ASD GML-3:HPC LF initially provided amorphous GML-3 at a 1:4 ratio, but during storage, GML-3 rapidly crystallized with loss of solubility, suggesting that it is undesirable to use HPC LF to create ASD.

## Figures and Tables

**Table 1:** Correlation coefficients according to Korsmeyer–Peppas model used for description of the dissolution mechanism of GML-3 from SD.

SD	*k* (min^−n^)	n	R^2^
GML-3:Soluplus^®^ (1:4)	0.358	0.253	0.998
GML-3:HPC LF (1:4)	0.627	0.111	0.980
GML-3:HPC EF (1:4)	0.842	0.205	0.995
GML-3:Soluplus^®^ (1:4) after 3 weeks (40 °C and 75%RH)	0.350	0.253	0.998
GML-3:HPC LF (1:4) after 3 weeks (40 °C and 75%RH)	0.155	0.406	0.995
GML-3:HPC EF (1:4) after 3 weeks (40 °C and 75%RH)	0.576	0.131	0.962

## Data Availability

The original contributions presented in this study are included in the article. Further inquiries can be directed to the corresponding author.
